# Intensity of hypertensive exposure in young adulthood and subclinical atherosclerosis in middle age: Evidence from the CARDIA study

**DOI:** 10.3389/fcvm.2022.959146

**Published:** 2022-12-07

**Authors:** Zhenyu Xiong, Jiaying Li, Yifen Lin, Xiaomin Ye, Peihan Xie, Shaozhao Zhang, Menghui Liu, Yiquan Huang, Xinxue Liao, Xiaodong Zhuang

**Affiliations:** ^1^Department of Cardiology, The First Affiliated Hospital, Sun Yat-sen University, Guangzhou, China; ^2^NHC Key Laboratory of Assisted Circulation, Sun Yat-sen University, Guangzhou, China; ^3^Guangdong Provincial Geriatrics Institute, Guangdong Provincial People's Hospital, Guangdong Academy of Medical Sciences, Guangzhou, China; ^4^Department of Ultrasonography, The First Affiliated Hospital, Sun Yat-sen University, Guangzhou, China; ^5^Center for Information Technology and Statistics, The First Affiliated Hospital, Sun Yat-sen University, Guangzhou, China

**Keywords:** coronary artery calcium (CAC), blood pressure, intima-media thickness (IMT), prognostic value, young adults (18–29 years)

## Abstract

**Background:**

Chronically high blood pressure (HBP) is a known risk factor for cardiovascular diseases. We measured the intensity of hypertensive exposure in young adults and calculated its prognostic significance for subclinical atherosclerosis in middle age.

**Methods:**

The Coronary Artery Risk Development in Young Adults (CARDIA) study enrolled 5,115 healthy black and white Americans who were 18–30 years old at baseline (1985–1986). The intensity of hypertensive exposure was calculated as the area under the curve (mm Hg × years) from baseline to year 15. Coronary artery calcium (CAC) was identified at years 15, 20, and 25, and intima-media thickness (IMT) was identified at year 20.

**Results:**

At baseline, the mean age was 40.1 years; 55.1% of participants were women, and 46.5% were black. After adjustment, cumulative systolic BP (SBP) was positively associated with CAC [hazard ratio (HR) = 1.23 (1.14, 1.32)] and IMT [β = 0.022 (0.017, 0.028)]. For CAC, the C-statistic for cumulative SBP was 0.643 (0.619, 0.667); compared to baseline SBP, the net reclassification index (NRI) of cumulative SBP was 0.180 (0.115, 0.256) and the integrated discrimination improvement (IDI) was 0.023 (0.012, 0.036). For IMT, the C-statistic for cumulative SBP was 0.674 (0.643, 0.705), the NRI was 0.220 (0.138, 0.305), and the IDI was 0.008 (0.004, 0.0012).

**Conclusion:**

Greater intensity of hypertensive exposure in early adulthood is associated with subclinical atherosclerosis in middle age and provides better prognostic value than baseline BP for early cardiovascular risk.

## Introduction

Chronically high blood pressure (HBP) is a major risk factor for morbidity and mortality worldwide (1–3). The 2017 American College of Cardiology/American Heart Association diagnostic criteria for hypertension (4) (systolic BP ≥ 130 mm Hg or diastolic BP ≥ 80 mm Hg) reclassify many people, especially young populations. This has increased the number of people with hypertension by 2–3 times (5), translating into an increased hypertensive burden in young adults worldwide.

Considering that BP is a dynamic risk factor, a single measurement is not enough to reflect the real intensity of hypertensive exposure over a long time, especially in young adulthood. Due to the proven associations of coronary artery calcium (CAC) and carotid artery intima-media thickness (IMT) with future cardiovascular disease (CVD) events (6–8), quantifying higher cumulative BP exposure during young adulthood may help in the early identification of populations at high risk for future cardiovascular events (9, 10).

Prior studies have shown that higher cumulative BP is a strong predictor of CVD events in middle-aged to older participants (11). However, it is uncertain whether higher cumulative BP exposure in young adulthood is associated with subclinical atherosclerosis, and whether the degree of cumulative exposure could provide better predictive value for CVD risk in middle age. In this study, we measured the association between the intensity of hypertensive exposure in young adults and the presence of subclinical atherosclerosis, as well as the association's prognostic value for cardiovascular risk, across 25 years of follow-up.

## Methods

### Participants

The Coronary Artery Risk Development in Young Adults (CARDIA) Study is a multicenter prospective study that recruited 5,115 healthy black and white young adults who were between the ages of 18 and 30 at baseline. Participants were enrolled from four US field centers (Birmingham, AL; Chicago, IL; Minneapolis, MN; and Oakland, CA) in 1985 and 1986 (12). After baseline examination (year 0), follow-up examinations were conducted at years 2, 5, 7, 10, 15, 20, 25, and 30; the retention rate across examinations was 72% at year 25. Data for the present study were extracted from years 0 through 25. All participants provided written informed consent at each study, and the Institutional Review Boards at each study site and coordinating center granted approval annually for all examinations (12).

We excluded participants who were without CAC examination (*n* = 152). We also excluded participants without any recorded baseline characteristics: i.e., BP (*n* = 15), body mass index (BMI) (*n* = 34), fasting glucose (FG) (*n* = 52), or total cholesterol (TC) (*n* = 3), as well as those whose race was not recorded as “black” or “white” (*n* = 12). After these exclusions, 3,403 participants were available for analysis. We further excluded those without any recorded data on intima-media thickness (*n* = 557) for further analysis (Supplementary Figure 1).

### Intensity of hypertensive exposure

BP was recorded at each visit in a quiet room after subjects had been seated for 5 min. Trained staff obtained three readings from the brachial artery at 1-min intervals using a random-zero sphygmomanometer (13).

We calculated the cumulative BP exposure from 0 to year 15 as mm Hg × year of each visit to represent long-term exposure to BP levels. We defined this BP product as each participant's accumulated exposure to BP (14, 15). We used the area under the BP curve over 15 years as a covariate in the univariable and multivariable analyses. Calculations were performed for systolic blood pressure (SBP) and diastolic blood pressure (DBP).

### Covariates

Standardized protocols for data collection were used across all examinations (12). All participants were required to fast for at least 12 h before each examination, as well as to avoid smoking or doing heavy physical activity for at least 2 h.

Information on age, sex, race, education, current smoking status, current drinking status, and medication history were self-reported. BMI was calculated as weight in kilograms divided by height in meters squared. TC and high-density lipoprotein cholesterol (HDL) were measured in fasting plasma samples and determined by enzymatic procedures. FG was assayed at baseline using the hexokinase ultraviolet method by American Bio-Science Laboratories (Van Nuys, CA).

### Coronary artery calcium

We obtained CAC measurements by computer tomography (CT) of the chest and determined it as either present or absent (CAC = 0 vs. CAC > 0) at years 15, 20, and 25 (16). Electron beam CT scanners (Chicago and Oakland sites) and multidetector CT scanners (Birmingham and Minneapolis sites) were used to obtain contiguous 2.5–3-mm-thick transverse images from the root of the aorta to the apex of the heart at years 15 and 20. Multidetector CT scanners (all sites) were used at year 25. Participants' CT images were transmitted to an independent reading center (Wake Forest University, Winston-Salem, NC) (16). Total CAC scores were calculated *via* image analysis by an improved Agatston method blinded to participant characteristics, with selected over-reading by an expert in cardiovascular imaging.

### Intima-media thickness

At year 20, Images of the left and right distal common carotid artery, carotid bulb, and proximal internal carotid artery were obtained by high-resolution B-mode ultrasonography using a standardized protocol. IMT was calculated from the average of the mean intima-media thicknesses of the internal, bulb, and common carotid near and far walls of each side (17). To assess the association between the intensity of BP exposure and IMT, we use IMT as both a continuous variable and a categorical variable.

### Statistical analysis

Descriptive statistics were used as means and standard deviations (SD) for continuous variables and as number (percentage) for categorical variables. Multivariable Cox regression models were used to evaluate the hazard ratio (HR) and 95% confidence interval (CI) for CAC and multivariable linear regression models, and logistic regression models were used to evaluate the β and odds ratio (OR) for IMT. We adjusted the above models according to baseline age, sex, race, education, current smoker status, current drinker status, body mass index, fasting glucose, total cholesterol, high-density lipoprotein, and antihypertensive medication use. Interactions by sex, race, current smoker status, current drinker status, and antihypertensive medication use were tested. The C-statistic, net reclassification index (NRI), and integrated discrimination improvement (IDI) were assessed.

A 2-sided *p* < 0.05 was considered statistically significant. All analyses were performed *via* SPSS software, version 25.0.

## Result

**Figure 1:**
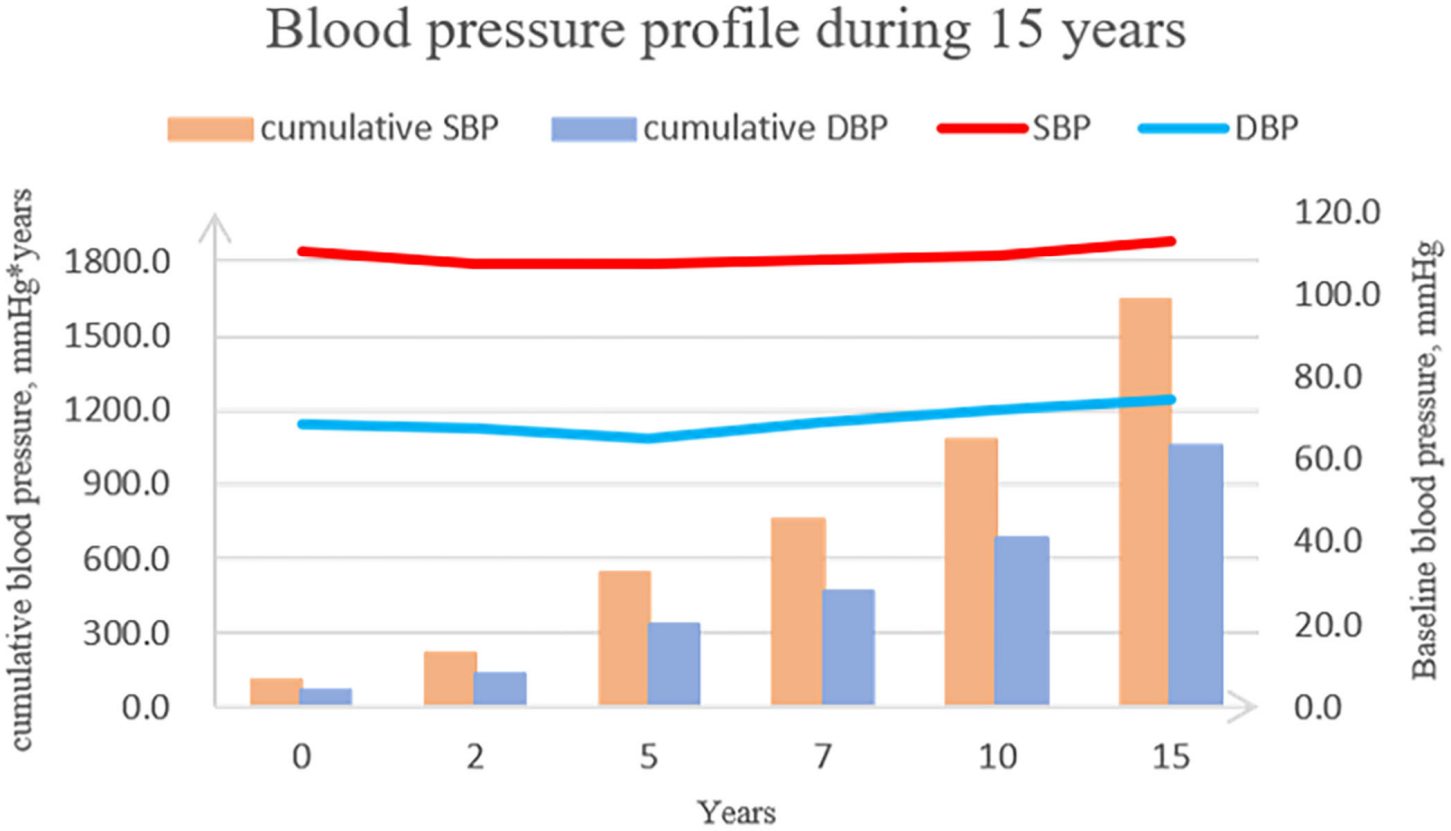
Blood pressure profile during 15 years.

Cohort characteristics at baseline (year 15) are described in Table 1. Among 3,403 participants, the mean (standard deviation) age was 40.1 (3.6) years; 55.1% were women, and 46.5% were black. Although the average BP of the population was similar across time, accumulated BP exposure accelerated steadily (Figure 1). Participants with higher cumulative BP exposure were more likely to be men, black, and current smokers, to have lower education levels and HDL, and to have higher BMI, SBP, DBP, FG, TC, and antihypertensive medication use.

**Table 1:** Baseline characteristics according to cumulative systolic blood pressure.

	**Quartile of cumulative SBP**				***P*-value**
**Characteristics**	**Q1**	**Q2**	**Q3**	**Q4**	
	**(≤ 1,532, *n* = 822)**	**(1,532–1,624, *n* = 852)**	**(1,624–1,726, *n* = 852)**	**(> 1,726, *n* = 877)**	
Age (years)	40.1 ± 53.7	40.1 ± 3.5	39.9 ± 3.6	40.2 ± 3.6	0.380
Women (%)	668 (81.3)	524 (61.5)	393 (46.1)	289 (33.0)	< 0.001
Black (%)	253 (30.8)	365 (42.8)	443 (52.0)	522 (59.5)	< 0.001
Education (years)	14.4 ± 2.0	14.0 ± 1.9	13.9 ± 2.0	13.5 ± 1.9	< 0.001
Current smoker (%)	136 (16.5)	177 (20.8)	182 (21.4)	228 (26.0)	< 0.001
Current drinker (%)	666 (81.0)	673 (79.0)	671 (78.8)	684 (78.0)	0.466
BMI (kg/mm^2^)	26.0 ± 5.5	28.1 ± 6.0	29.2 ± 6.1	30.9 ± 6.4	< 0.001
SBP (mmHg)	99.7 ± 7.2	108.2 ± 7.2	115.1 ± 9.0	128.8 ± 15.3	< 0.001
Cumulative SBP (mmHg*year)	1,463.1 ± 53.6	1,580.3 ± 27.3	1,673.4 ± 29.0	1,838.8 ± 108.3	< 0.001
DBP (mmHg)	66.4 ± 7.9	71.4 ± 7.9	76.1 ± 9.2	83.8 ± 12.3	< 0.001
Cumulative DBP (mmHg*year)	949.9 ± 71.2	1,019.4 ± 68.8	1,080.2 ± 79.0	1,058.8 ± 119.8	< 0.001
FG (mg/dl)	81.6 ± 13.0	84.3 ± 14.4	87.5 ± 21.7	92.7 ± 27.7	< 0.001
TC (mg/dl)	179.4 ± 32.1	183.8 ± 33.8	186.1 ± 36.9	189.2 ± 38.7	< 0.001
HDL (mg/dl)	54.9 ± 14.3	51.1 ± 14.2	49.4 ± 14.1	47.1 ± 14.2	< 0.001
Antihypertensive medication (%)	3 (0.4)	15 (1.8)	57 (6.7)	178 (20.3)	< 0.001
CAC (%)	142 (17.3)	197 (23.1)	244 (28.6)	375 (42.8)	< 0.001

Data are presented as mean (SD) or % as appropriate.

BMI, body mass index; SBP, systolic blood pressure; DBP, diastolic blood pressure; FG, fasting glucose; TC, total cholesterol; HDL, high-density lipoprotein cholesterol; CAC, coronary artery calcium.

During the 25-year follow-up, 985 participants (28.1%) developed CAC. The association between cumulative BP and CAC is shown in Table 2. In adjusted Cox models, cumulative BP was associated with CAC [HR = 1.23 (1.14, 1.32) for cumulative SBP and HR = 1.16 (1.08, 1.24) for cumulative DBP]. In the sensitivity analysis, we quartered cumulative BP, and the higher quartile of cumulative SBP and DBP were associated with CAC (both *p* for trend < 0.001).

**Table 2:** Association between intensity of hypertensive exposure and coronary artery calcium.

	**Quartile of cumulative blood pressure HR (95% CI)**				**P for trend**	**Per SD increment**
	**Q1**	**Q2**	**Q3**	**Q4**		**HR (95% CI)**
Baseline SBP						
Model 1	Reference	1.41 (1.15, 1.72)	1.69 (1.38, 2.08)	2.50 (2.07, 3.01)	< 0.001	1.37 (1.30, 1.44)
Model 2	Reference	1.13 (0.92, 1.39)	1.24 (1.01, 1.53)	1.49 (1.21, 1.82)	< 0.001	1.17 (1.01, 1.25)
Cumulative SBP						
Model 1	Reference	1.39 (1.12, 1.72)	1.79 (1.45, 2.20)	2.93 (2.42, 3.56)	< 0.001	1.50 (1.42, 1.59)
Model 2	Reference	1.05 (0.85, 1.31)	1.15 (0.92, 1.44)	1.51 (1.21, 1.88)	< 0.001	1.23 (1.14, 1.32)
Baseline DBP						
Model 1	Reference	1.27 (1.05, 1.54)	1.40 (1.15, 1.70)	1.97 (1.64, 2.35)	< 0.001	1.31 (1.23, 1.38)
Model 2	Reference	1.01 (0.84, 1.23)	1.00 (0.81, 1.21)	1.25 (1.03, 1.52)	0.02	1.13 (1.06, 1.21)
Cumulative DBP						
Model 1	Reference	1.44 (1.16, 1.78)	1.95 (1.59, 2.38)	2.66 (2.19, 3.22)	< 0.001	1.42 (1.34, 1.51)
Model 2	Reference	1.20 (0.97, 1.49)	1.34 (1.09, 1.66)	1.48 (1.19, 1.83)	< 0.001	1.16 (1.08, 1.24)

HR, hazard ratio; CI, confidence interval; SD, standard deviation; SBP, systolic blood pressure; DBP, diastolic blood pressure.

Model 1 was unadjusted, Model 2 was adjusted for baseline age, sex, race, education, current smoker, current drinker, body mass index, fasting glucose, total cholesterol, high-density lipoprotein and antihypertensive medication use.

The association between cumulative BP and IMT is shown in Table 3. In adjusted linear models, cumulative BP was associated with IMT [β = 0.022 (0.017, 0.028) for cumulative SBP and β = 0.008 (0.003, 0.014)]. For the sensitivity analysis, we defined the 90th percentile of IMT as abnormal IMT. In adjusted logistic models, cumulative SBP was associated with abnormal IMT [OR = 1.36 (1.20, 1.54)]. Furthermore, we quartered cumulative BP, and the higher quartile of cumulative SBP was associated with abnormal IMT (*p* for trend < 0.001) (Supplementary Table 1).

**Table 3:** Association between intensity of hypertensive exposure and intima-media thickness.

	**Intima-media thickness** β **(95% CI)**	
	**Model 1**	**Model 2**
Baseline SBP	0.038 (0.033, 0.043)	0.019 (0.014, 0.024)
Cumulative SBP	0.042 (0.037, 0.047)	0.022 (0.017, 0.028)
Baseline DBP	0.027 (0.022, 0.032)	0.009 (0.004, 0.014)
Cumulative DBP	0.031 (0.026, 0.036)	0.008 (0.003, 0.014)

CI, confidence interval; SE, standard error; SBP, systolic blood pressure; DBP, diastolic blood pressure.

Model 1 was unadjusted, Model 2 was adjusted for baseline age, sex, race, education, current smoker, current drinker, body mass index, fasting glucose, total cholesterol, high-density lipoprotein and antihypertensive medication use.

We also performed subgroup analyses for sex, race, current smoker status, current drinker status, and antihypertensive medication use (Supplementary Figure 2). We did not find interactions among these characteristics.

The predictive performance of the intensity of hypertensive exposure for subclinical atherosclerosis is shown in Table 4. For CAC, the C-statistic for cumulative SBP was significantly increased [0.643 (0.619, 0.667)] compared to baseline SBP [0.612 (0.587, 0.636)]. The NRI and IDI of cumulative SBP were significantly improved compared to the use of baseline SBP as a reference [0.180 (0.115, 0.256) for NRI and 0.023 (0.012, 0.036) for IDI]. For IMT, the C-statistic for cumulative SBP was significantly increased [0.674 (0.643, 0.705)] compared to baseline BP [0.658 (0.626, 0.690)]. The NRI and IDI of cumulative SBP were significantly improved compared to the use of baseline SBP as a reference [0.220 (0.138, 0.305) for NRI and 0.008 (0.004, 0.012) for IDI].

**Table 4:** Predictive and discriminatory performance of intensity of hypertensive exposure for subclinical atherosclerosis.

	**Coronary artery calcium**			**Intima-media thickness** > **90%**		
	**C-statistic**	**Category-free NRI (SE)**	**IDI (SE)**	**C-statistic**	**Category-free NRI (SE)**	**IDI (SE)**
Baseline SBP	0.612 (0.587, 0.636)	Reference	Reference	0.658 (0.626, 0.690)	Reference	Reference
Cumulative SBP	0.643 (0.619, 0.667)	0.180 (0.115, 0.256)	0.023 (0.012, 0.036)	0.674 (0.643, 0.705)	0.220 (0.138, 0.305)	0.008 (0.004, 0.012)
Baseline DBP	0.591 (0.565, 0.616)	Reference	Reference	0.614 (0.579, 0.649)	Reference	Reference
Cumulative DBP	0.633 (0.609, 0.656)	0.147 (0.082, 0.234)	0.020 (0.010, 0.031)	0.618 (0.584, 0.652)	0.073 (−0.012, 0.158)	0.004 (0.001, 0.007)

CI, confidence interval; SE, standard error; NRI, net reclassification index; IDI, integrated discrimination improvement; SBP, systolic blood pressure; DBP, diastolic blood pressure.

## Discussion

In this large prospective cohort study with a long-term follow-up of 25 years, we found that a higher intensity of hypertensive exposure during young adulthood was independently associated with higher subclinical atherosclerosis risk in middle age. Furthermore, cumulative BP provides better prognostic value for early cardiovascular risk compared to baseline BP.

According to the 2017 Hypertension Clinical Practice Guidelines, over 45% of American adults have hypertension (1, 5). Nevertheless, most of our understanding about BP comes from single measurement data in cross-sectional studies (18, 19). The present study described the overall BP profile during young adulthood (Figure 1). Mean BP fluctuated within a small range around the normal level, while cumulative BP increased gradually over time. This suggests that cumulative BP measurement could better reflect the real severity of BP over a long follow-up period. Our study found that the incremental prognostic value of using cumulative BP is an improvement over single baseline BP measurement for both CAC and IMT. Thus, calculating cumulative BP, such as with a smartwatch collecting daily blood pressure readings, may contribute to BP management and early CVD risk stratification for young adults.

To our knowledge, this study is the first to reveal the independent association between cumulative blood pressure exposure in young adults and subclinical atherosclerosis –in midlife, suggesting that more attention should be paid to blood pressure management in young adults. Prior studies investigating the dynamic evolution of blood pressure focused mainly on elderly individuals (20–23). With a growing number of young people reclassified as hypertensive according to the new BP guidelines, many young adults now face an increased hypertensive burden, which could cause them to experience risks such as subclinical atherosclerosis earlier in time. Therefore, assessing the association between early hypertensive exposure and subclinical atherosclerosis (as represented by CAC and IMT) with data from the CARDIA study, with its high proportion of seemingly healthy participants [as is reflected in the low (7.4%) prevalence of hypertensive medication use], is meaningful. A cross-sectional study in Korea found that higher BP categories were positively associated with prevalent CAC in a young, low-risk population (24). Our study features a 25-year follow-up cohort with strict, high-quality examinations at each visit. Community and primary health care interventions may be useful for promoting the longitudinal decline of BP in young adults, thus mitigating the deleterious effects of hypertension in later life (2, 25, 26). Previous studies have reported that BP profile is associated with CVD events (27, 28) and cardiac structure and function (14). Our study extends the area of cumulative BP exposure to early prevention of cardiovascular events in young adulthood. Furthermore, our study represents a first in identifying the prognostic value of cumulative BP for subclinical atherosclerosis risk in young adults.

The deleterious effects of hypertension vary depending on the duration and intensity of high BP (29). An imbalance among endothelial dysfunction, inflammation, and reactive oxygen species results in a vicious cycle of increased cardiovascular disease risk (30). The cumulative effects of insults to the blood vessel lead to a pro-hypertensive environment. In the early stages of elevated BP, stage 1 hypertension, or in young and middle-aged hypertensive patients, effective control of BP leads to maintaining balance, with little organ involvement. Inversely, poor BP control may initiate secondary mechanisms (e.g., the renin-angiotensin system and endothelial and vascular smooth muscle–related mechanisms), causing aggravated BP levels, atherosclerosis (31), and finally, target organ damage. Thus, lifestyle management or antihypertensive medication use may easily control BP in earlier stages but fail to maintain a favorable BP level in the end stage. In this study, we found that cumulative BP exposure had better prognostic value compared to single baseline BP for the presence of subclinical atherosclerosis; it may therefore help young adults control blood pressure better and thereby prevent later CVD events.

### Strengths and limitations

The strengths of this study include its prospective design and 25 years of follow-up in people from young adulthood to middle age; the general good health of the study population; the reduplicative measurements of both exposures and outcomes; the abundant data on potential confounders measured along with the diagnosis of hypertension; a high retention rate; and the standardized BP data collection protocols and strict quality control in the CARDIA study (1).

However, the current study also has several limitations: First, treatment of hypertension may have influenced the results of our findings; however, in this young cohort, our findings remained significant after adjusting for antihypertensive medication use. Second, the subclinical outcomes, rather than the CVD events, in this study may not directly translate into clinical practice. However, the assessment of subclinical atherosclerosis in young adults should be considered. Third, residual confounding from measured or unmeasured variables remains a possibility, though we have adjusted for risk factors as much as possible.

In conclusion, we found that cumulative BP in young adults was independently associated with subclinical atherosclerosis in middle age, and that cumulative BP provides better prognostic value for early cardiovascular risk compared to baseline BP. This finding highlights the prognostic value of using cumulative BP and may enable a greater focus on early blood pressure management in young people.

## Data availability statement

Publicly available datasets were analyzed in this study. This data can be found at: https://www.cardia.dopm.uab.edu/ (CARDIA study).

## Ethics statement

The studies involving human participants were reviewed and approved by IEC for clinical research and animal trials of the First Affiliated Hospital of Sun Yat-sen University and the CARDIA study. The patients/participants provided their written informed consent to participate in this study.

## Author contributions

ZX, JL, XL, and XZ: research idea and study design. ZX, JL, and YL: data analysis/interpretation. ZX, SZ, and XZ: statistical analysis. ZX, XY, JL, and ML: manuscript drafting. XL and XZ: data acquisition, guarantors of this work and, as such, had full access to all the data in the study and took full responsibility for the integrity of the data and the accuracy of the data analysis. PX and YH: manuscript revision. All authors contributed important intellectual content during manuscript writing or revision, and read and approved the final manuscript.
