# Supplementary material for: Intensity of hypertensive exposure in young adulthood and subclinical atherosclerosis in middle age: Evidence from the CARDIA study
Source: Front Cardiovasc Med. 2022 Dec 7;9:959146. doi: 10.3389/fcvm.2022.959146 (PMC9768548; doi:10.3389/fcvm.2022.959146)
Supplement: Supplementary file 1 [file Data_Sheet_1.PDF]

Supplementary Figure 1. Flow chart of study.

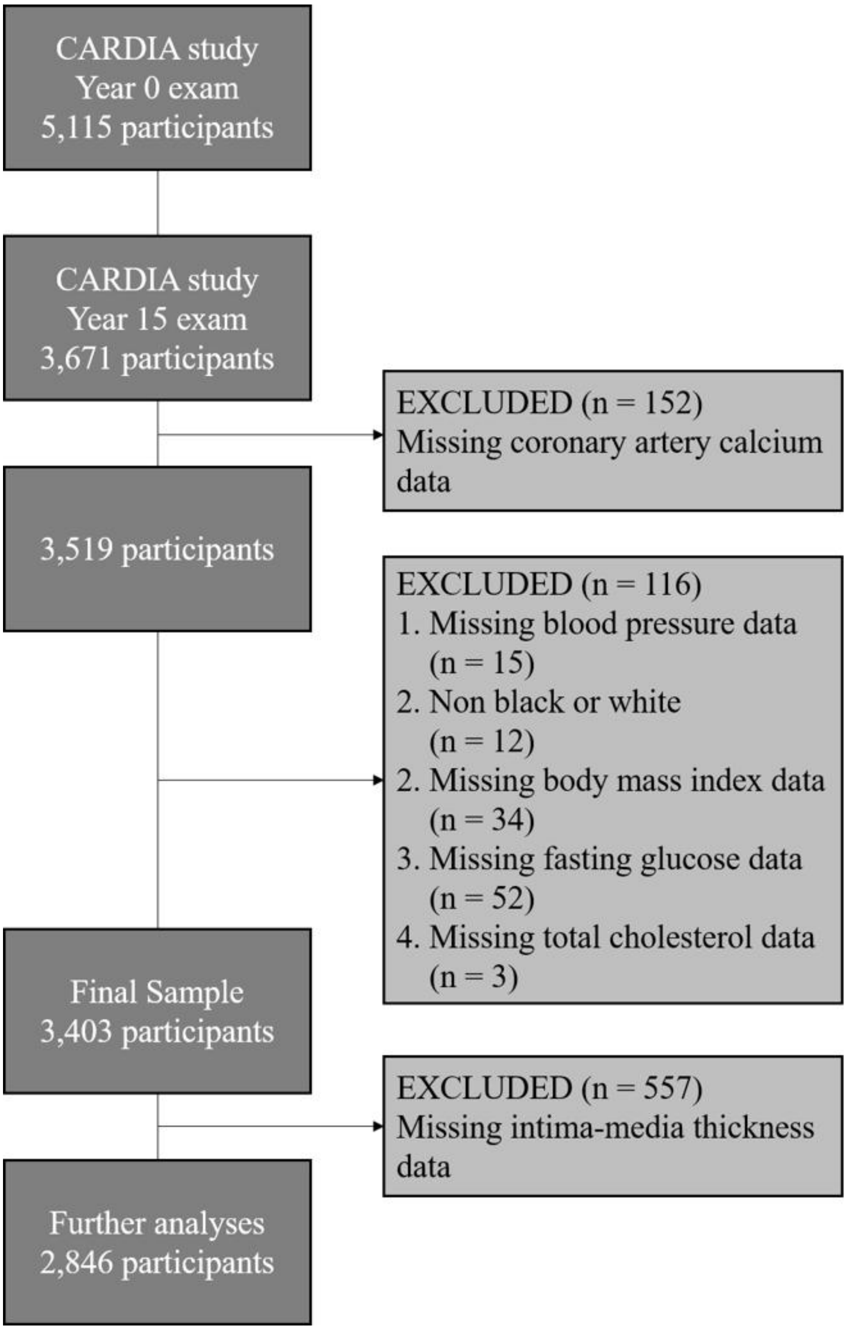

Supplementary Figure 2. Forest plots of subgroup analyses.

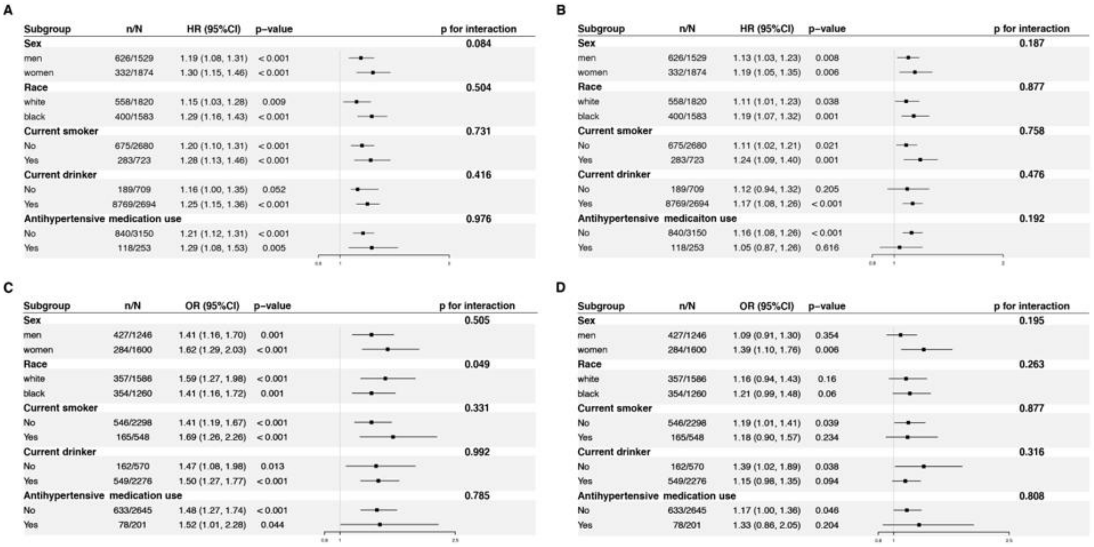

A: subgroup analyses of cumulative SBP and CAC.

B: subgroup analyses of cumulative DBP and CAC.

C: subgroup analyses of cumulative SBP and IMT.

D: subgroup analyses of cumulative DBP and IMT.

Model was adjusted for baseline age, sex, race, education, current smoker, current drinker, body mass index, fasting glucose, total cholesterol, high-density lipoprotein and antihypertensive medication use.

Supplementary table 1. Association between intensity of hypertensive exposure and intima-media thickness > 90%.

	Quartile of cumulative blood pressure OR (95% CI)				P for trend	Per SD increment OR (95% CI)
	Q1	Q2	Q3	Q4		
Baseline SBP						
Model 1	Reference	1.32 (0.86, 2.04)	2.78 (1.87, 4.15)	3.66 (2.50, 5.37)	< 0.001	1.61 (1.44, 1.78)
Model 2	Reference	1.02 (0.65, 1.59)	1.93 (1.27, 2.95)	2.00 (1.31, 3.05)	< 0.001	1.36 (1.20, 1.54)
Cumulative SBP						
Model 1	Reference	2.06 (1.28, 3.31)	2.85 (1.80, 4.49)	5.78 (3.77, 8.88)	< 0.001	1.77 (1.58, 1.98)
Model 2	Reference	1.58 (0.97, 2.57)	2.03 (1.25, 3.28)	3.30 (2.03, 5.37)	< 0.001	1.49 (1.29, 1.72)
Baseline DBP						
Model 1	Reference	1.30 (0.88, 1.92)	1.88 (1.28, 2.77)	2.83 (1.97, 4.06)	< 0.001	1.45 (1.29, 1.62)
Model 2	Reference	0.97 (0.64, 1.46)	1.28 (0.85, 1.93)	1.59 (1.07, 2.37)	0.005	1.21 (1.06, 1.38)
Cumulative DBP						
Model 1	Reference	1.52 (1.01, 2.27)	1.59 (1.07, 2.37)	2.92 (2.02, 4.22)	< 0.001	1.53 (1.36, 1.71)
Model 2	Reference	1.29 (0.84, 1.95)	0.99 (0.65, 1.51)	1.42 (0.94, 2.17)	0.209	1.19 (1.04, 1.38)

Abbreviation: OR = odds ratio, CI = confidence interval, SD = standard deviation, SBP = systolic blood pressure, DBP = diastolic blood pressure

Model 1 was unadjusted, Model 2 was adjusted for baseline age, sex, race, education, current smoker, current drinker, body mass index, fasting glucose, total cholesterol, high-density lipoprotein and antihypertensive medication use.
